# A protocol for a cluster-randomized controlled trial testing an empowerment intervention to prevent sexual assault in upper primary school adolescents in the informal settlements of Nairobi, Kenya

**DOI:** 10.1186/s12889-019-7154-x

**Published:** 2019-06-27

**Authors:** Clea Sarnquist, Jennifer Lee Kang, Mary Amuyunzu-Nyamongo, Gabriel Oguda, Dorothy Otieno, Benjamin Mboya, Nancy Omondi, Duncan Kipkirui, Michael Baiocchi

**Affiliations:** 1 Stanford University School of Medicine, Stanford, CA USA; 2 African Institute for Health and Development, Nairobi, Kenya; 3 Ujamaa-Africa, Nairobi, Kenya

**Keywords:** Violence against woman and girls, Adolescent, Kenya, Protocol, Intervention

## Abstract

**Background:**

Sexual violence against adolescents is prevalent worldwide and results in significant physical and mental injuries as well as loss of economic and personal potential. Urban informal settlements such as those around Nairobi, Kenya have been shown to have especially high incidences of violence. Research has shown that empowerment interventions for female adolescents can reduce sexual assault. However, these interventions have had limited testing in urban informal settlements, with young adolescents, or in coordination with complementary programs for male adolescents.

**Methods/design:**

This study was a two-arm, parallel, cluster-randomized trial testing a combination of a previously-tested girls’ intervention, IMPower, and a newly revised boys’ intervention, Source of Strength. Clusters were defined as schools within the informal settlements; participants were adolescent girls and boys in class 6, generally between the ages of 10–14 at baseline. Data collection began in January 2016 and continued through December 2018. The primary outcome was the change in incidence of self-reported sexual assault among girls from baseline, compared to a life skills standard of care intervention. Secondary outcomes included experiences of physical and emotional violence, as well as determining the effects of the intervention on self-efficacy, self-esteem, and gender attitudes and beliefs, and how those effects led to changes in experience of sexual assault. For the primary outcome and several of the secondary outcomes, we used an intention to treat estimand.

**Discussion:**

This was the first randomized controlled trial with longitudinal follow-up of an empowerment self-defense approach to violence prevention for adolescents in informal settlements. The large size and rigorous design supported analysis to understand multiple subgroup experiences in the hypothesized reduction in sexual assault. The study was also unique in its focus on young (10–14 years of age) adolescents and in engaging both boys and girls in separate but coordinated curriculums. The focus on a highly vulnerable and understudied population will make it a significant contribution to the literature on violence prevention.

**Trial registration:**

Clinical Trials.gov #NCT02771132. Version 3.1 registered May 2017, first participant enrolled January 2017. Retrospectively registered.

## Background

An estimated 120 million girls worldwide – 1 in 10 – are raped or sexually assaulted by the age of 20 years [1]. In sub-Saharan Africa, 16 to 59% of adult women report having ever been sexually assaulted, with many assaults occurring during adolescence or childhood [2]. The 2010 Kenya Violence Against Children Survey (VACS) showed that 11% of girls and 4% of boys aged 13–17 experienced some type of sexual violence in the 12 months prior to the survey, and 3 out of 10 girls and 2 out of 10 boys reported at least one episode of sexual violence before the age of 18 [3]. Adolescents living in the informal settlements, or slum communities, of Nairobi, Kenya also report high rates of sexual assault, between 8 and 25% annually [4–6], depending on age. Furthermore, cross-sectional data from nine countries confirm that adolescent girls and young women are at higher risk of intimate partner violence (IPV), which may include sexual assault, than older women [7]. Sexual assault of young people results in a wide range of negative health and social outcomes, including physical injury or disability, unplanned pregnancies, psychological trauma, and school drop-out [8–13].

### Knowledge gaps

To-date, most sexual assault prevention interventions have been evaluated in high-income countries [14]. Limited research has been conducted about sexual assault either among adolescents or in informal settlements, and very minimal research is available at the intersection of young people in informal settlements, despite them being a large, high-risk, and growing population. Informal settlements are an important setting to study, as they are growing quickly worldwide, and have high rates of violence, infectious disease, and other threats to human health and well-being [15].

A few interventions have shown promise in low- and lower-to-middle income countries (LMIC), although generally with older populations or in dis-similar settings. The most relevant of these is the combined Stepping Stones and Creating Futures intervention, which was implemented in informal settlements around Durban, South Africa in 2014 [16, 17]. Although the study population consisted of older adolescents and young adults (18–30 years of age), the environmental characteristics of the settlements are similar to the setting where we plan to conduct this trial: high poverty rates, inadequate access to clean water and sanitation, poor quality of housing, and overcrowding. That preliminary study found improvements in gender attitudes and mental health as well as a decrease in controlling behaviors by men as a result of the intervention. A large, randomized controlled trial of this is currently underway in similar communities in South Africa, the findings of which will be important to compare to results from the study described here.

A larger and more rigorous trial was the 2014 evaluation of SASA!, which showed that community mobilization improved gender norms and reduced intimate partner violence (IPV) incidence and acceptability among both adult women and men in in Kampala, Uganda [18]. The SHARE study, also in Rakai, Uganda, included adolescents from 15 years of age (although it also included adults up to 59 years old); it combined HIV care and community mobilization to improve IPV-related behaviors, decrease physical IPV, and decrease HIV incidence [19]. Another successful trial was IMAGE, which reduced IPV among older adolescents and adults in South Africa through a combination intervention incorporating gender issues and microfinance. The original IMAGE trial, however, did not include adolescents under 18 years of age and was conducted in a rural setting [20]. Amongst the age group that is the focus on this study, adolescents 10–14, there have been some promising studies in high-income countries, although most have had short follow-up periods and/or only query changes in skills, not actual assault. Examples include an empowerment self-defense based intervention in New Zealand [21] and a skills and knowledge program in California [22].

Our research group has also conducted preliminary studies of the interventions described in this paper. In 2012 and 2013, we evaluated similar girls’ empowerment and boys’ transformation interventions, separately, in secondary schools (student ages 13–20) in several Nairobi settlements. The intervention for girls was based on an empowerment self-defense approach or “resistance” approach, which has only been studied in high-income countries, [23–27] and almost always with positive results. The primary outcome in the girls’ studies was sexual assault incidence. The interventions were successful in significantly reducing incidence by 38–63% compared to the girls receiving the standard-of-care (SOC) [5, 28]. The boys’ intervention reduced negative gender norms; these changes were sustained one year later. It also increased the likelihood of boys intervening on behalf of girls in risky situations [29]. In 2014, we researched a pilot expansion of these stand-alone interventions into a dual-gender program, where boys and girls received complementary, parallel, but separate interventions. That expansion also involved randomization and enrolling a younger population, between 10 and 14 years of age (in upper primary school). In that study, we found the intervention caused a decrease of 3.7 percentage points in annual incidence of rape from a baseline of 7.3% [6]. The three early studies were all observational with convenience sampling, and the randomized trial was an open-cohort design without individual longitudinal follow-up. In addition, the maximum follow-up period in these studies was 10 months, with the ability to collect data on covariates limited due to resource constraints.

This study fills significant gaps in the knowledge base, specifically about adolescents in urban settlements. It is a replication study of our previous RCT, but also improves upon our previous study design by providing longitudinal follow-up, a longer follow-up period (24 months), the ability to collect covariates that may help predict or explain outcomes, and a revised intervention for boys that focuses on positive masculinity as well as gender norms. It was also the largest study ever of an empowerment/self-defense based approach for rape prevention among adolescents.

### Current study

This protocol tested the effectiveness of an intervention combining the successful girls’ intervention, IMPower, and a significantly revised boys’ intervention, Source of Strength (SOS). As these are challenging populations and environments, with very limited resources or infrastructure, and high levels of poverty and crime, they required innovative partnerships and methods in order to conduct a rigorous randomized-controlled trial. These approaches are described below.

## Methods/design

### Study design

This was a two-arm, parallel, cluster-randomized trial with clusters defined as schools within the settlements. Data collection began in January 2016 and continued through December 2018.

The study was conducted in six informal settlements in Nairobi: Kibera, Mukuru, Huruma, Dandora, Kariobangi, and Korogocho. For the purposes of the study, given the small number of schools from Kariobangi, Kariobangi schools were considered part of the Dandora settlement. Local partners included three non-governmental organizations, No Means No Worldwide (NMNW), Ujamaa Africa, and the African Institute for Health and Development (AIHD). NMNW created the intervention, Ujamaa implemented the intervention, and AIHD was the Nairobi-based research partner for the evaluation component of the study. Stanford University was the independent, external evaluator of the intervention.

The primary outcome was the change in incidence of self-reported sexual assault among adolescent girls from baseline, comparing the IMpower and SOS interventions to a SOC intervention. Secondary outcomes included determining the impact of the interventions on self-efficacy, self-esteem, gender attitudes and beliefs, and experiences of physical and emotional violence. We also measured episodes of perpetrating violence and bystander behaviors among adolescent boys. In follow-up surveys, we collected data on acceptability of the program and how often adolescents recall using specific skills from the program. All of these data were collected using quantitative surveys. We also collected process data at follow-up on what components of the intervention the adolescents found useful and what the adolescents suggest changing about the intervention in the future. Stakeholder meeting of teachers, head teachers, and officials from the Kenyan Ministry of Education were held before each round of data collection to answer questions and gain buy-in.

We also collected qualitative data through in-depth interviews with both girls and boys. These focused on the experiences and understanding of violence in the communities, as well as characteristics of implementation to gain a deeper understanding of successes and failures of the intervention.

### Inclusion/exclusion criteria

Included schools could be operated by the government, non-governmental organizations (NGOs), or private enterprises, but must not have received the intervention in the previous 3 years (the intervention has been previously taught in several of these communities). Participating schools must operate within, or draw most of their student population from, one of the informal settlements listed above. Due to the dynamic nature of school recruitment and deployment of the study, some schools withdrew post-randomization but pre-deployment. One school dropped out post-randomization, and it was replaced with a different school.

Inclusion/exclusion criteria for adolescents included: enrolled in class six (sixth grade) in a sampled school (see sampling description below), able to communicate in at least one of the study languages (English or Kiswahili), and not suffering from a significant mental deficit (learning difficulty, mental illness or substance abuse) that would impair their ability to consent to participation in the trial (as determined by school and study staff).

### Randomization

Clusters: Stanford researchers used pre-trial characteristics of the schools to create matched sets of schools. Matching before randomization was used to reduce discrepancies in important covariates values across arms of the trials arising during randomization [30]. The school-level covariates considered in the matching were the number of girls and boys (separated by sex) in classes 5–8 (separated by class), school-level average standardized test scores, number of toilets (by sex), teachers per capita, settlement, school sponsor type (i.e., government, private, religious), and physical building materials (wall material, roof material). Within each matched set, one school was assigned to the standard of care (SOC) arm and one school to the girls’ intervention (Table 1). Each school within the set had an equal probability of being assigned to either of the two arms. Randomizations resulting in any pairwise standardized difference outside of the range [− 0.1, 0.1] were rejected and the randomization was rerun. Due to logistics of deploying the intervention, three cohorts of schools were randomized. The cohorts had no intentional distinctions, other than when the schools were recruited. As the intervention is a training program, it is not possible to blind schools or trainers to their study arm.

**Table 1:** Summary of randomization of the 108 schools randomized to treatment or SOC

Baseline covariate	Mean of Treatment Schools	Mean of SOC Schools	Pooled Standard Deviation	Standardized Mean Difference
Number of girls in class 6	78	74	56	0.08
Number of boys in class 6	72	73	55	−0.02
Total number of students in school	571	560	410	0.03
Total number of teachers in school	15	15	8	0.01
Teachers per capita schoolwide	53.2	49.9	42.1	0.08
Total number of rooms where classes are taught	11	9	5	0.25
School’s mean score on prior year’s standardized test	270	269	40	0.02
Missing indicator for prior year’s standardized test	6%	9%	25%	−0.12
Number of toilets per capita in school	0.05	0.04	0.03	0.08
Percent of schools in Dandora	24%	15%	40%	0.22
Percent of schools in Huruma	32%	18%	44%	0.33
Percent of schools in Kibera	15%	9%	34%	0.17
Percent of schools in Korogocho	12%	26%	39%	−0.38
Percent of schools funded by government	68%	59%	50%	0.18
Percent of schools funded privately	18%	24%	42%	−0.14
Percent of schools funded by religious institution	3%	9%	29%	−0.20
Percent of schools with walls made of stone	38%	50%	50%	−0.24
Percent of schools with walls made of blocks	18%	15%	37%	0.08
Percent of schools with walls made of tin sheets	9%	18%	34%	−0.26
Percent of schools with walls made of concrete	12%	9%	29%	0.10
Percent of schools with roof made of iron sheets	82%	79%	38%	0.08

Individuals: Among smaller schools (*n* < 40, within sex, in grade 6), all pupils in the school participated in the baseline survey. For logistical reasons, in large schools, adolescents were randomly selected using a lottery method (picking colored beads from an opaque bag) to participate in the surveys. The larger schools tended to be sampled to sizes less than 70 per sex.

### Sample size

We powered the study to distinguish the intervention from the SOC group based on the sexual assault prevention outcome. Based on our 2013–14 pilot study, we assumed that baseline annual incidence in similar informal settlements, in this age group, is 7.3% [6]. From this prior study, we also estimated the interclass correlation to be approximately 0.001.

We calculated sample size to achieve 80% power of detecting approximately a 40% decrease in the odds of sexual assault as reported by the participants when comparing the intervention group to SOC group (technically, calculated as a risk difference from 0.07 to 0.044). We took into account a school-level dropout of 25% and a participant-level dropout of 25% over the course of the study. Calculations to determine the sample size were run using the *CRTSize* package in R, function *n4props()* [31]. Given the above assumptions, we estimated that we needed a minimum of 45 schools for each arm of the trial, with at least 40 girls in each school.

We also recruited male participants into the study to complete surveys in order to better understand their experiences of violence in this setting. As this is an exploratory aim, this study is not powered on boys’ outcomes. The specific number of boys studied in each school was maximized given restrictions due to funding; we randomly sampled between 5 and 15 boys per school and included all of the sampled schools that had boys enrolled in class 6.

For the qualitative data, we purposively sampled 20 girls and ten boys from one participating school in the Dandora neighborhood to participate in in-depth interviews. This sampling approach was taken in order to allow for deep understanding of one of the study communities.

### Intervention

Curricula for both interventions, IMPower and SOS, were developed specifically by NMNW to target younger adolescents in Nairobi [32, 33]. Both girls’ and boys’ interventions were taught in six two-hour sessions held weekly on school property. Sessions in the intervention included role-plays, facilitated discussions, and verbal and physical skills practice.

The girls’ intervention focused on empowerment, avoiding risky situations, verbal skills, and physical self-defense. Specific session topics included: (1) building rapport and self-esteem and providing definitions and objectives; (2) personal awareness, self-efficacy, boundaries, and assertive communication skills; (3) verbal and physical defense skills; (4) review of verbal and physical skills and skills practice using bags and mitts; (5) de-escalation and negotiation, and more advanced defense techniques for use in instances such as multiple or armed attackers; (6) review of all previous sessions and a discussion of sexual assault and harassment experiences.

The boys’ training focused on promoting gender equality, developing positive masculinity, and teaching safe and effective bystander intervention techniques [29]. This intervention was different from a previously-studied version of the intervention in order to incorporate learnings from implementing the previous intervention. Specific session topics included: (1) building rapport and develop awareness about gender interactions; (2) personal awareness, learning assertive body language and verbal response; (3) intervention, verbal and physical defense skills; (4) defining and understanding sexual consent, valid consent, causes and myths about rape, de-escalation and negotiation techniques; (5) responsibility for one’s actions and behaviors, practice using intervention skills; (6) reviewing key concepts; reinforcing skills through role-plays, and public commitments to utilize new skills.

Both female and male trainers facilitated the sessions with a trainer:student ratio of approximately 1:15. Trainers were chosen through an intensive interview and vetting process to ensure that they were respected members of their communities and had a background in health improvement. All trainers received extensive instruction by expert facilitators, and participated in mock interviews and field training exercises conducted outside of the study area. Trainers were required to pass written and oral examinations, as well as a physical skills demonstration before teaching the curriculum. The training and field exercises combined took approximately 1 year to complete before trainers were allowed to teach classes as the main trainer.

### Standard of care

The SOC group received a one-time 1.5–2-h life skills class that is supported by the Kenyan Ministry of Education and includes a wide range of topics, including sexual assault, sanitation, food safety, and personal rights. All school-aged children who attend an accredited school typically receive this curriculum. Thus, participants in the intervention arm are likely to also receive the SOC at some point in their educational careers. These sessions were taught by trained NMNW facilitators on school property for consistency.

After the completion of the study and post-final data collection, NMNW trained facilitators provided a sample of schools in the SOC arm of the study with the intervention.

### Timeline

Because Ujamaa had a core cadre of very experienced trainers who had undergone extensive preparation, as described above, we did not want to threaten the quality of the intervention by bringing in many new trainers. Therefore, we divided the schools into three cohorts, so each group could be trained by the existing experienced trainers. The cohorts were rolled-out between January–September of 2016, with 12 and 24 month follow-up between January–September of 2017 and 2018, respectively.

### Quantitative data collection and analysis

#### Survey development

There are very few validated scales available for this group (10–14 year old girls) regarding sexual abuse, and variables on the pathways to sexual abuse. Therefore, we drew from a variety of sources in creating our surveys. In order to measure the primary outcome, change in incidence of self-reported sexual assault among girls from baseline, we created an index based on several variables. Key surveys that informed these questions included the Kenya VACS [34] and surveys from the Stepping Stones project in South Africa [16, 35]. These items include both experiences of IPV (for example, “In the past 12 months, how many times has your current or a previous boyfriend physically forced you to have sex when you did not want to?” and “In the past 12 months, how many times has your current or a previous boyfriend used threats or intimidation to get you to have sex when you did not want to?”) as well as sexual violence by non-partners (“In the past 12 months, how many times has a man who is NOT your boyfriend forced or persuaded you to have sex against your will?” and “In the past 12 months how many times was there an occasion when you agreed to have sex with one man or boy and one or more others who you had not agreed to have sex with forced you to have sex with them as well?”).

Validated, widely used scales were more readily available for some of the secondary outcomes. Specifically, we used the “Self-Efficacy Questionnaire for Children” [36], for self-efficacy, and the “Rosenburg Self-Esteem Scale” [37], for self-esteem. For experiences of social and emotional violence, we relied on the VACS and Stepping Stones surveys. For gender norms and relations, some questions were removed from existing surveys and modified for this population during piloting, and others were created based on the field team’s knowledge of this population, then modified during piloting. We also collected data on acceptability of the program and how often adolescents recall using specific skills from the program.

#### Baseline analysis

The baseline data collected were analyzed to provide insights into the prevalence and characteristics of violence in these settlements. Summaries of: (i) demographic information of participants, (ii) rates of intimate partnerships, (iii) baseline rates of violence (non-GBV), (iv) baseline rates of GBV (non-rape), (v) baseline rate of rape, (vi) distribution of count of rapes (i.e., some participants will be raped many times and some not at all, we will summarize this distribution), (vii) distributions of self-efficacy and self-esteem, and (viii) distribution of perpetrators. Further, we used a principal stratification approach to estimate the relationship between hypothesized intermediate effects – self-efficacy, alcohol use, gender norms, and self-esteem – and rape [38]. The analysis of hypothesized intermediate effects informed the discussion of causal pathways from the intervention to the outcome (related to “structural equation modeling” approaches, or “mediation analysis”).

#### Outcome analysis

We used an intention to treat (ITT) estimand to analyze the primary outcome of change in incidence of self-reported sexual assault among adolescent girls from baseline. An instrumental variable (IV) analysis was also proposed as a secondary analysis to estimate the effectiveness of the intervention [39], but was not tenable because trainee-attendance records were not well-maintained.

We estimated the effect of the intervention on the rate of sexual assault on the participant level using generalized linear mixed models (GLMM), with each participant’s survey being a unique observation in the model. Note that the analyses were stratified by sex – models were run separately for girls and for boys. The outcome of interest was binary and thus will be modeled using a logistic link function. Missing covariate values were addressed using multiple imputation models.

The estimand of interest was a repeated measures effect, the change of incidence of sexual assault between the prior twelve-month period at baseline as compared to the twelve-month period between the first follow-up period and the final follow-up period on the participant level. In our GLMM, repeated measure effects were added to the model for individual girl, school, and matched set. Fixed effects were added for period (i.e., baseline, first follow-up and end of study), intervention level, and an interaction for period and intervention level. The coefficient for the interaction term between period and intervention level was our estimate of interest. The GLMM was chosen in order to facilitate comparisons with other interventions in the What Works to Prevention Violence Against Women and Girls consortium. A GEE model of the main outcome was performed as a sensitivity analysis.

### Dropout – individual-level and school-level

All efforts were made to prevent loss of participants at follow-up points. In the pilot study, though we did not track individuals using unique identifiers, retention was high (> 80%) at follow-up (roughly 10 months post-intervention). In the current study, unique identifiers were used, and participants were tracked longitudinally. To deal with observational units that dropped out at the endline data collection period, the analysis used baseline covariates to construct an inverse probability-of-missing weighting to address units that dropped out at the endline. A sensitivity analysis of the outcome analysis addressed school-level if schools drops out post-intervention; if schools dropped out pre-intervention, this will be noted for consideration of the external validity of the study. The goal of the sensitivity analyses was (1) to properly estimate standard errors and (2) to bound the size of bias that the loss of a school might have on our estimate. Sensitivity analyses included using a weighted, resampling approach of the observed data to form “stand-in” schools for the lost schools.

We conducted secondary analyses using similar approaches to estimate rates of change in other outcomes as detailed above. Fisher’s exact tests were used to assess (i) changes in skills used, (ii) perpetrators, and (iii) disclosure rates and identify of persons disclosed to.

### Qualitative data collection and analysis

The goal of the qualitative component was to gain in-depth understanding of the adolescents’ experiences of violence and safety in their schools, homes, and communities. The sample included 20 girls and 10 boys, and they were followed longitudinally, with data collected through in-depth interviews at baseline, and 12 and 24 months post-intervention. Interviews were conducted after school in private spaces by trained, experienced, sex-matched, interviewers from AIHD. Table 1 contains the domains explored in the interviews.

Interviews were not recorded due to concerns about safety and security in the informal settlement settings; rather the interviewer took notes, which were transcribed immediately following each interview. Transcripts were de-identified and sent electronically to Stanford University, where they were analyzed using a thematic approach [40]. Weekly calls with Kenyan investigators included discussion of the interpretation of the data based on their unique knowledge of this setting and population.

### Ethics, consent, and permissions

As previously discussed, the study protocol was reviewed and approved by both the Stanford University Institutional Review Board and the Kenya Medical Research Institute (KEMRI). Significant protocol changes went undergo review and approval of these bodies before implementation. The protocol is registered with ClinicalTrials.gov (Protocol #NCT02771132).

Nonetheless, this study involves highly sensitive subject matter, experiences of physical and sexual assault, in an adolescent population with significant economic and social vulnerability. Many steps were taken to minimize the possibility of harm from this study. Because study subjects were under the age of 18, written informed assent was sought by AIHD field staff. Written informed consent was also sought from parents. One feature of this study to try to improve the assenting process was that the assenting was done in the first visit with the adolescents, and the survey was done in the second visit. This decision was made to ensure ample time for each the assenting process and the survey, realizing that young adolescents have limited attention span, and that we only had about 1.5 h available for each study visit. Therefore, rather than rush the assenting process, an entire session was devoted primarily to ensuring that potential participants understood and felt comfortable with giving assent.

Parental informed consent was obtained using an opt-out approach. This approach was utilized, and approved by both the Stanford and KEMRI IRBs for several reasons, including: many of the children in the informal settlements lack stable parents or legal guardians, those parents and guardians may be very difficult to reach, and there is a possibility that a parent or other family member/caregiver may be perpetrating, or know about, the violence. Information about the study was sent home with children for their parents. Head teachers also informed the students, parents and the school committee members about the study. Parents/guardians of children sampled to participate in the study could have chosen to opt-out of participation at these meetings or during follow-up and this was recorded by signature or thumb-print. All parents were given a chance to ask questions about the study and were informed that their child’s participation in the study is completely voluntary and that they could have withdrawn from the study at any time. A data monitoring committee was not used because there was no interim data analysis to review per funder requirements. In addition, since this was a one-time intervention, the intervention could not have been stopped based on interim data analysis. Study staff were trained on Stanford University policies for identifying and reporting adverse events.

There is a potential for psychological distress to participants in any research on gender-based violence. The World Health Organization and the Sexual Violence Research Initiative have developed guidance on safety in conduct of research in this area [41, 42]. Research has shown that if these guidelines are followed there are minimal psychological risks attached to survey research on gender-based violence [43, 44]. This study included the guidance from these documents in its design and implementation. However, in cases where participants demonstrated distress or report being emotionally impacted by the research questions or intervention, they were referred to local sources for additional support. Participants who reported experiencing sexual assault were referred to Sexual Assault Survivors Anonymous (SASA) support groups run by Ujamaa and NMNW, as well as to medical services, as needed. Additional referral resources include the Center for Rights Education and Awareness (which provides integrated services for survivors and legal support), the Gender Violence Recovery Centre at Nairobi Women’s Hospital, Medecins Sans Frontieres’ Centre for Victims of Sexual Violence in Kibera, and Childline Kenya (a national toll-free telephone and web-based helpline for children).

The intervention also had specific elements intended to reduce potential harm to participants, possibly increasing their ability to recognize and avoid risky situations, defend themselves against sexual assault and physical violence, and improve their self-efficacy, communication, and negotiation skills. These skills may be protective against not only sexual and physical violence but also may have positive long-term benefits in empowering adolescents to make informed choices about their reproductive, sexual, and physical health [5, 26, 45, 46]. The results of our previous pilot trials, previously described, showed that girls reported significantly less sexual assault and boys reported significantly higher rates of bystander intervention to prevent sexual assault, benefiting not only the individual participants but also members of their communities [5, 6, 28, 29]. The intervention was also offered to a sample of SOC schools in 2018. Finally, research has shown that while single-session sexual violence prevention interventions might be ineffective, teaching self-defense skills has not been shown to increase risk of harm [47]. Instead, recent evaluations of empowerment self-defense interventions, albeit among female college students in North America, have shown that women who receive these interventions are significantly less likely to experience assault than women in control groups [23, 26, 48].

### Confidentiality

Unique IDs, which did not contain personal information, were created at the outset of the study and recorded on all forms. Each participant had a unique ID assigned, and that ID was be the only piece of identifying information on all data collection tools. The surveys were given in small groups of two to four girls with one interviewer. Each girl received her survey in a manila folder, and was encouraged to keep the survey in the folder during the entire period to minimize the ability of classmates to see her answers. At the end of the survey, manila folders were closed and slipped into a larger, opaque folder which was tied shut and put inside a backpack that the research team leader carried. Thus there was very minimal chance for classmates or the enumerators to see the responses.

Data was entred only using unique IDs, and survey response data was stored in a password protected file “survey file”, which linked the responses to the unique ID only. A separate, password-protected file “identification file” that linked the unique-person IDs to the real-world person linked the unique-ID to demographic information. The identification file was be maintained at Stanford.

All data were entered and maintained in a REDCap database. REDCap is a secure, web-based application designed to support data capture for research studies that was developed by a multi-institutional consortium and is supported at Stanford University [49]. REDCap is designed to comply with U.S. regulations on protection of health information and privacy, and utilizes a secure, Stanford-specific server.

## Discussion

This was the first randomized controlled trial with individual-level longitudinal follow-up of an empowerment self-defense approach to violence prevention for adolescents in a low-income setting, to the best of our knowledge. The study was large, with over 90 clusters (schools) participating and thousands of young people enrolled. This sample size was sufficient to support rigorous analysis of causal pathways between the intervention and the prevention of violence. Furthermore, several innovative study design approaches, previously discussed, were used: (1) in the randomization process to ensure balance between the SOC and intervention groups (Table 2), and (2) to maximize distance between the SOC and intervention sites, as we expect that study participants may teach skills from the intervention to their family and friends.

**Table 2:** Qualitative Survey Domains

Description of neighborhood and interpersonal relationships.	
Exploring the definition/understanding of “safety” and “health”.	
Description safety and safety concerns in the context of the community, school, and home.	
Description of safety and safety concerns in the context of relationships.	
Exploring the definition of “violence” and describing violence in the context of personal experiences.	
Discussing coping and defense strategies related to violence and safety.	
Exploring the experience with the intervention (follow-up surveys only).	

The population targeted in this research, young adolescents aged 10–14 years living in urban informal settlements, has been poorly studied regarding their experiences of violence, and has rarely been the focus of violence prevention programs. Nonetheless, they are an essential age group to reach. First, they are already experiencing high rates of sexual and physical violence, so have immediate need for prevention approaches [50]. Second, older adolescents (about ages 15 and up) are at high risk for sexual violence [5, 6, 28], so prevention efforts can be maximized by reaching young people before they enter this period. Third, research shows that negative gender biases and discrimination develop as late as early adolescence, so an intervention in this period may be especially well timed to prevent the development of negative beliefs that may lead to risky behavior [51].

Understanding patterns of violence, and options for prevention, in informal settlements is crucial to global health. It is estimated that one billion people currently live in such settlements, and that number is expected to rise considerably in the coming decades [52]. Informal settlements are characterized by lack of services and infrastructure, as well as insecurity, and often have high rates of violent crime. As the numbers of people living in these communities continues to increase, evidence-based interventions that engage community members to improve social, health, and economic conditions are urgently needed. It is possible that the intervention studied here will prove to be one such evidence-based prevention approach.

Finally, this study is innovative in including both boys and girls in violence prevention. Dual-sex approaches continue to be less common than single-sex approaches, but there is increasing recognition that community-level violence reduction requires thoughtful engagement of men and boys as well as women and girls. An example of a dual-sex approach that has shown effectiveness is bystander intervention, where young men and women can be taught to recognize high-risk situations and intervene to possibly stop an assault [53]. In addition, research shows that gender inequitable masculinity is related to men perpetrating sexual assault, and gender transformative approaches requires the engagement of both sexes [54, 55]. These points may be especially important in adolescent populations, where, as discussed above, gender norms and beliefs may not yet be fully formed, and thus may be more malleable.

### Limitations

Major study limitations include the focus on in-school youth and the inability to tease out the differential effects of the programs for boys versus girls. It is likely that focusing on adolescents in school excludes the young people at highest risk of sexual violence. In Kenya, this limitation is somewhat mitigated because primary school is free. Thus, the net primary school attendance ratio is 72% for boys and 75% for girls [56]; these are considerably higher, especially for girls, than in many settings where school fees are a barrier to attendance. Nonetheless, ~ 25% of young people are out of school, and hidden fees such as uniforms and supplies, as well as expectations about caring for younger siblings and performing household chores, often prevent adolescents, especially girls, from attending school periodically. Focusing in schools also makes it difficult to know if this intervention could work with an out-of-school population, as socializing teachers and school headmasters to violence-recognition and prevention efforts may also have prevention effects. Furthermore, since school could opt-into the study, there may be bias in the types of schools that chose to participate. In addition, the study is not designed to be able to segregate the effect of the boy’s intervention from that of the girls. As the primary outcome is sexual assault, it would be difficult to tease these out without a larger, more resource-intensive multi-arm trial. Given that the field is generally moving towards the thoughtful inclusion of men and boys, and that some of arguments for inclusion are especially salient for young adolescents, a multi-arm trial did not seem necessary.

## Conclusion

This study has the potential to add to the growing body of evidence on preventing violence, especially sexual assault. It is unique in its focus on a highly-vulnerable population, young adolescents living in informal settlements, and the empowerment self-defense approach.

## Data Availability

Due to the highly sensitive nature of these data on sexual assault, as well as the age of the subjects (10–14 at baseline), these data will be available only through request to the authors, who will vet requests to be certain that appropriate IRB approvals and data safety guidelines are in place.

## Abbreviations

AIHD: African Institute for Health and Development
GBV: Gender Based Violence
GEE: Generalized Estimating Equations
IIT: Intention to Treat
IPV: Intimate Partner Violence
IV: Instrumental Variable
KEMRI: Kenya Medical Research Institute
LMIC: Low- and Lower-to-Middle Income Countries
NGO: Non-Governmental Organizations
NMNW: No Means No Worldwide
SOC: Standard of Care
SOS: Source of Strength
VACS: Violence Against Children Surveys
