# Expression of Concern to: A protocol for a cluster-randomized controlled trial testing an empowerment intervention to prevent sexual assault in upper primary school adolescents in the informal settlements of Nairobi, Kenya

**DOI:** 10.1186/s12889-021-10263-4

**Published:** 2021-01-27

**Authors:** Clea Sarnquist, Jennifer Lee Kang, Mary Amuyunzu-Nyamongo, Gabriel Oguda, Dorothy Otieno, Benjamin Mboya, Nancy Omondi, Duncan Kipkirui, Michael Baiocchi

**Affiliations:** 1 Stanford University School of Medicine, Stanford, CA USA; 2 African Institute for Health and Development, Nairobi, Kenya; 3 Ujamaa-Africa, Nairobi, Kenya

**Expression of Concern to: BMC Public Health 19, 834 (2019)**

**http://orcid.org/10.1186/s12889-019-7154-x**

The Editor is issuing an editorial expression of concern to alert readers that after publication, concerns were raised regarding this article [1]. The article presents the original study protocol and states that ‘The surveys were given in small groups of two to four girls with one interviewer’, which is what was originally planned. However, after publication it has come to light that in the actual study this interviewer to participant ratio was much higher, which is not reflected in the article, even though it was published after the conclusion of the study. Post publication peer review concluded that the higher ratio could affect the results, in that it may have caused underreporting of sexual assault incidents. The actual ratio is under dispute among the authors. Authors Clea Sarnquist, Jennifer Lee Kang, Mary Amuyunzu-Nyamongo, Gabriel Oguda and Michael Baiocchi disagree with this Expression of Concern. Authors Benjamin Mboya, Nancy Omondi and Duncan Kipkirui agree with this Expression of Concern. Author Dorothy Otieno has not responded to correspondence regarding this Expression of Concern.
